# Anti-leishmanial and Anti-inflammatory Agents from Endophytes: A Review

**DOI:** 10.1007/s13659-019-00220-5

**Published:** 2019-09-28

**Authors:** Rufin Marie Kouipou Toghueo

**Affiliations:** Antimicrobial and Biocontrol Agents Unit (AmBcAU), Laboratory for Phytobiochemistry and Medicinal Plants Studies, Department of Biochemistry, Faculty of Science, University of Yaoundé I, P.O. Box 812, Yaoundé, Cameroon

**Keywords:** Anti-leishmanial, Anti-inflammatory, Endophytes, Bioactive metabolites

## Abstract

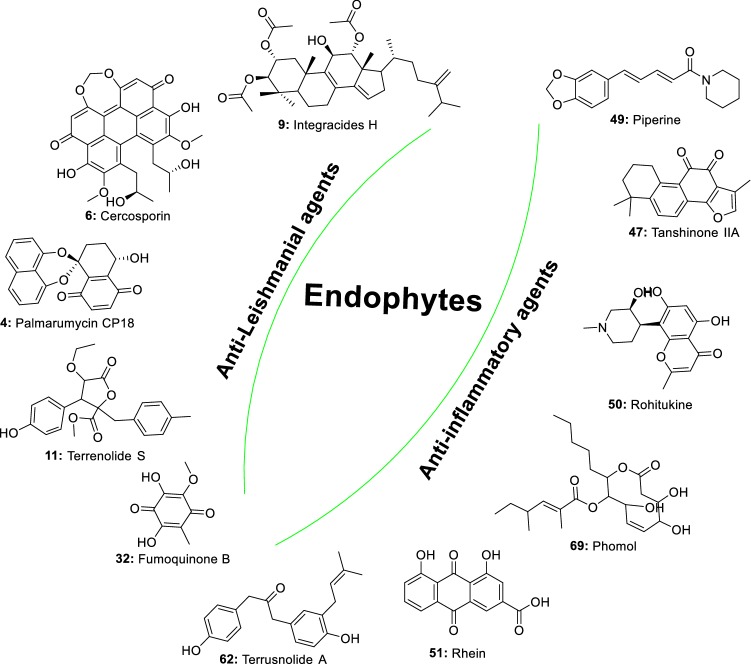

## Introduction

Natural products have and will continue to offer exceptional chemical diversity with a wide biological activities spectrum. They have been so far the most promising sources for drug discovery and development [1, 2]. According to Newman and Cragg [3] and Katz and Baltz [4], over 50% of all agents currently in clinical use are either natural products or natural product derived. The use of these products as medicines is ancient since for centuries, health practices have incorporated the medicinal properties of products from diverse sources, particularly microbial species. The modern discovery of penicillin and streptomycin led to the recognition of microbial natural products as one of the most powerful and prolific sources for drug lead discovery over the past seven decades [5]. Indeed, the rates of discovery for new drugs dramatically dropped since the year's nineties because many pharmaceutical companies stopped their natural products programmes in profit of high-throughput screening of synthetic chemicals [4].

Fortunately, recent years have seen a regain of interest in exploring natural products and leading pharmaceutical companies are currently screening microbial natural extracts for the development of high-throughput libraries [3, 6]. Moreover, there is also a growing attraction toward products from other underexplored sources [7], particularly microorganisms from higher plants [8]. Living inside plants without causing any symptoms of diseases, endophytes are recognized to play a significant role in affecting the secondary metabolite types and the quantities synthesized through a complex microbial-host interaction [9]. Endophytes are capable of synthesizing bioactive compounds that are used by plants for defence against pathogens [10]. These compounds produced by endophytes have proven to be useful for novel drug discovery. Most of the bioactive compounds isolated from endophytes are known to have functions of antibiotics, immunosuppressants, anticancer agents, biological control agents, and so forth [11, 12]. The structural diversity of their secondary metabolites offers impressive and continuous pools for medicinal chemistry applications to accelerate the discovery of new drugs against several diseases. Over the last decade, secondary metabolites isolated from endophytes from medicinal plants have been investigated for their anti-leishmanial and anti-inflammatory activities. We are summarizing in this review previous literature on active metabolites isolated from endophytes, hoping to provide a useful guide for future investigations aiming to identify lead compounds needed to accelerate the discovery of new drugs against leishmaniases and inflammatory diseases.

## Leishmaniases: Leads Discovery from Endophytes

### The Context

Leishmaniases regroup diseases caused by *Leishmania* parasites. Historically, these diseases have been affecting mankind for a very long time as evidenced by the discovery of potteries from pre-Colombian and the 1000-year-old sculls with shreds of evidence of leishmaniases [13]. Presently, more than 20 *Leishmania* species are known to infect humans via the female phlebotomine sandflies. Looking at their geographical distribution and the clinical manifestations of the infection, these parasites are among the most diverse human pathogens. Clinically, we have visceral leishmaniasis (VL) which is the most serious form of the disease, cutaneous leishmaniasis (CL), the most common and mucocutaneous leishmaniasis [14]. Worldwide, 350 million people are at risk of acquiring these diseases. In 2017, 97 countries and territories were endemic for leishmaniasis. 20792 new cases were reported among which 22145 (94%) occurred in seven countries including Brazil, Ethiopia, India, Kenya, Somalia, South Sudan and Sudan [14]. In disease-endemic areas, many infected individuals remain asymptomatic for prolonged durations [15]. However, in a state of compromised immunity such as in HIV infection, the progression from the asymptomatic to symptomatic visceral or cutaneous forms can substantially increase [16, 17]. The HIV patients co-infected with VL are particularly difficult to treat and are also associated with increased rates of relapse, mortality and high transmission [18, 19]. Presently, treatments for leishmaniasis are limited to a few drugs including pentavalent antimonials, amphotericin B, paromomycin, and miltefosine, the only oral treatment available. However, serious drawbacks including prolonged treatment duration, parenteral administration, low tolerability, teratogenicity, treatment failures, the requirement for cold storage and high cost have been reported [20, 21]. Moreover, the efficacy of these drugs varies from one geographical location to another [22]. Therefore, the identification of a low-cost, safe, effective, oral, short-course drug and suitable for use in pregnancy is desperately needed [23]. Regarding the chemical structure of drugs currently used against this infectious disease, it is obvious that the *Leishmania* spp. can be inhibited by compounds belonging to diverse chemical classes. This is of interest since endophytes are reputed to produce diverse structurally novel chemical scaffolds with outstanding activity. Therefore, researches are currently underway to identify and evaluate potential new candidates produced by plant endophytes.

### Antileishmanial Metabolites Produced by Endophytes

**Fig. 1:**
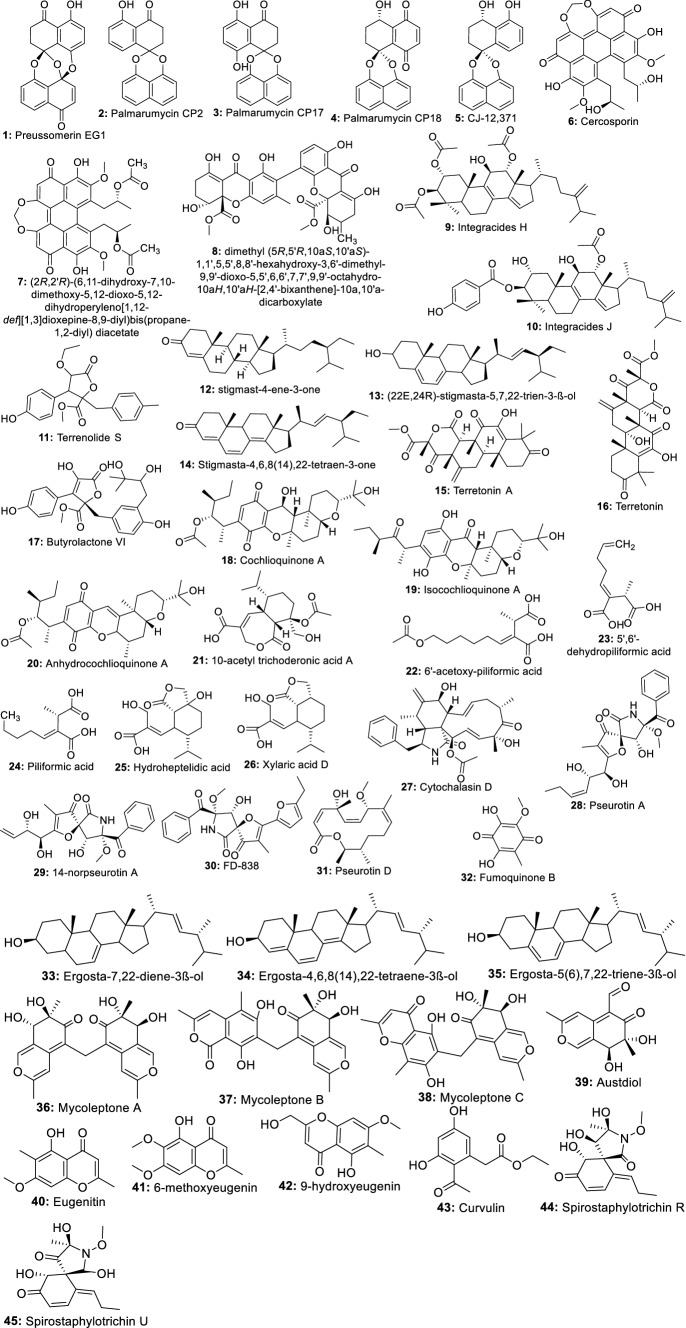
Metabolites active against *Leishmania* spp. isolated from endophytic fungi

The recent years have seen the increased interest of scientists to investigate endophytes for their ability to produce compounds with a cidal effect against *Leishmania* spp. This rush driven by the need for new treatment has led to the identification of several active metabolites with the potential to constitute a good starting point for novel anti-leishmanial drug discovery. Indeed, as a part of massive natural product drug discovery projects, extracts from more than 2700 endophytic fungi were screened for their activity against several parasites including *Leishmania donovani*. The results show that up to 17% of isolates were active and the most potent belonged to Nectriaceae, Trichocomaceae and Mycosphaerellaceae families [24]. In another investigation, one hundred and twenty-one endophytic fungi isolated from four Brazilian plants were tested against *Leishmania amazonensis*. Extracts from eleven fungi (9%) were able to inhibit the growth of *L. amazonensis *with IC_50_ values ranging from 4.6 to 24.4 µg/mL. The potent endophytic fungi were identified as belonging to the genera *Alternaria, Arthrinium, Cochliobolus, Colletotrichum, Penicillium, Fusarium*, and *Gibberella* [25]. These results indicate that endophytic fungi extracts may contain bioactive prototype molecules against *Leishmania* parasites and suggest that future chemical investigation may lead to the identification of potentially active compounds (Fig. 1).

Indeed, the bioassay-directed fractionation of extracts from the fungus *Edenia* sp. led to the isolation of preussomerin EG1 (**1**), palmarumycin CP2 (**2**), palmarumycin CP17 (**3**), palmarumycin CP18 (**4**), and CJ-12,371 (**5**). These compounds showed activity against *Leishmania donovani* amastigote, with IC_50_ values of 0.12, 3.93, 1.34, 0.62, and 8.40 µM, respectively [26]. In a later investigation, Ortega et al. [27] also showed that palmarumycin CP18 (4) can inhibit *L. donovani* in a macrophage with an IC_50_ value of 23.5 µM. From this finding, we can suggest that palmarumycins have the ability to cross the macrophage membrane to inhibit the intramacrophage parasites. Therefore, further optimization of these compounds may provide more potent lead molecules. Likewise, cercosporin (**6**) and its derivative (**7**) displaying very high potency (IC_50_ of 0.46 and 0.64 µM respectively) against *Leishmania donovani* were also identified among the six compounds isolated from a crude extract of *Mycosphaerella* sp. nov. strain F2140, an endophyte of *Psychotria horizontalis* [28]. Another compound, a new ergochromone derivative (**8**), very potent against *L. donovani* (IC_50_ 0.87 μM) was also isolated from an extract of *Purpureocillium lilacinum* endophyte from roots of *Rauvolfia macrophylla* [29]. Integracides H (**9**), and Integracides J (**10**) isolated from *Fusarium* sp. endophyte of *Mentha longifolia* were also reported to inhibit *Leishmania donovani* with IC_50_ values of 4.75 and 3.29 μM respectively [30]. Seven other compounds including a new butenolide derivative, terrenolide S (**11**), along with stigmast-4-ene-3-one (**12**) (22*E*,24*R*)-stigmasta-5,7,22-trien-3-β-ol (**13**), stigmasta-4,6,8(14),22-tetraen-3-one (**14**), terretonin A (**15**), terretonin (**16**) and butyrolactone VI (**17**) isolated from extract of *Aspergillus terreus*, an endophyte from the roots of *Carthamus lanatus* were also reported to inhibit *Leishmania donovani* with IC_50_ ranged from 11.24–87.34 µM [31]. All these compounds highly potent with diverse chemical structures, constitute attractive scaffolds for future medicinal chemistry to synthesize new candidates to fill the pipeline for the development of new more effective drugs against visceral leishmaniasis, the most severe form mostly caused by *L. donovani*.

Using the same bio-guided approach to investigated the active extract of *Cochliobolus* sp.UFMGCB-555 isolated from the plant *Piptadenia adiantoides* J.F. Macbr, Campos et al. [32] isolated cochlioquinone A (**18**) and isocochlioquinone A (**19**), two potent compounds against *L. amazonensis* with IC_50_ of 1.7 and 4.1 µM. Similarly, a fractionation of crude extract of *Cochliobolus sativus* selected as the most potent from a screening of endophytic fungi from leaves of *Vernonia polyanthes* led to the identification of cochlioquinone A, isocochlioquinone A and anhydrocochlioquinone A (**20**) as the active ingredients [33]. These compounds active in the micromolar range are also small molecules, ideal for further optimization through medicinal chemistry. Recently, in a preliminary screening to search potential antileishmanial compounds, Cota et al. [34] identified ethyl acetate extract of the fungus *Nectria pseudotrichia*, an endophyte of *Caesalpinia echinata* as promising against *Leishmania braziliensis*. The fractionation led to seven compounds including 10-acetyl trichoderonic acid A (**21**), 6′-acetoxy-piliformic acid (**22**), 5′,6′-dehydropiliformic acid (**23**), piliformic acid (**24**), hydroheptelidic acid (**25**), xylaric acid D (**26**), and cytochalasin D (**27**). All the compounds exhibited activity with compounds **21**, **22**, and **25** being more active, with IC_50_ values of 21.4, 28.3, and 24.8 µM respectively. Martínez-Luis et al. [35] previously reported the very good activity of compounds, pseurotin A (**28**), 14-norpseurotin A (**29**), FD-838 (**30**), pseurotin D (**31**), and fumoquinone B (**32**) isolated from endophytic *Aspergillus* sp. strain F1544.

Twenty metabolites including one new isochromene derivative named nigrosphaerin A were isolated from an extract of the endophytic fungus *Nigrospora sphaerica* and tested for antileishmanial activity. Only three including compounds **33**, **34** and** 35** showed moderate antileishmanial activity with IC_50_ values of 30.2, 26.4 and 36.4 μg/mL, respectively [36]. Similarly, three new azaphilones named mycoleptones A, B, and C (**36**–**38**) along with austdiol (**39**), eugenitin (**40**), 6-methoxyeugenin (**41**), and 9-hydroxyeugenin (**42**), were also isolated from extract of *Mycoleptodiscus indicus*, a fungus associated with the medicinal plant *Borreria verticillata*. When tested for their antileishmanial activity, all the seven compounds exhibited moderate to weak potency [37]. In a more recent study, active extracts from endophyte *Bipolaris* spp. were investigated. Curvulin (**43**), spirostaphylotrichins R (**44**) and U (**45**) were identified as the main components. These compounds exhibited moderate antileishmanial activity with IC_50_ values ranging from 70–84.2 μg/mL [38]. An endophytic fungus *Fusarium tricinctum* isolated from fruits of *Hordeum sativum* Jess was reported to produce well-known antibiotics compounds Enniatins A, A1, B, B1, B2 and Q exhibiting moderate antileishmanial activity [39].

Recently, Alves et al. [40] showed that lipases produced by three endophytic fungi namely *Emericella nidulans*, *Dichotomophtora portulacae* and *D. boerhaaviae* could also inhibit *Leishmania amazonensis* with growth inhibition ranging from 39.65 to 98.13%. In an earlier report, ten secondary metabolites including a new polyketide derivative koninginin H were isolated from *Emericella nidulans* by Tarawneh et al. [41] and none of these compounds show potency against *Leishmania* spp. Altogether, these findings open a possibility of exploring endophyte beyond secondary metabolites as a source for new drugs.

## Inflammation

### Brief Background

As pieces of evidence in ancient medical texts, inflammation has been known to humankind for thousands of years. However, its clinical symptoms were clearly defined in the first century AD by the Roman doctor Cornelius Celsus [42, 43]. The inflammation is the response of the immune system triggered by a variety of factors, including pathogens, damaged cells, toxic compounds or exposure to radiation. The etiology of inflammation can be infectious or non-infectious. These factors have been reported to induce acute and/or chronic inflammatory responses in several organs of the body including the heart, pancreas, liver, kidney, lung, brain, intestinal tract and reproductive system, potentially leading to tissue damage or disease depending on the causing agent [44]. For instance, chronic inflammation has been reported as a consequence of leishmanial infection. Studies have shown a high production of pro-inflammatory cytokines such as IL-4 and IL-10 in patients with VL [45–47]. According to Lima-Junior et al. [48], to resist the host cell and replicate in macrophages, these parasites produce nitric oxide (NO), a pro-inflammatory molecule. Many other reports also demonstrated the inflammatory profiles particularly mediated by IL-8, IFN-γ, TNF-α, and IL-6 cytokines, in patients suffering from VL [49]. Overall, if not treated, chronic inflammation can lead to the development of multiples diseases like stroke, chronic respiratory diseases, heart disorders, cancer, obesity, and diabetes [50–52]. These chronic inflammatory diseases are the most significant cause of death in the world [53]. For the treatment, multiples drugs are available, among which non-steroidal anti-inflammatory drugs, are the most commonly prescribed. These medications are particularly useful not only because of their high effectiveness, but they also help decrease pain, and control swelling [54]. However, they are highly toxic and known for their multiple adverse effects, including gastrointestinal bleeding, cardiovascular side effects, and nephrotoxicity to name just a few [55]. Therefore, new effective drugs with fewer toxic effects are needed for better management of inflammatory diseases. The potential of endophytes to produce such active ingredients has been reported and will constitute the discussion of this section.

### Endophytes as Sources of Novel Anti-inflammatory Agents

#### Plant Secondary Metabolites Produced by Endophytic Fungi

**Fig. 2:**
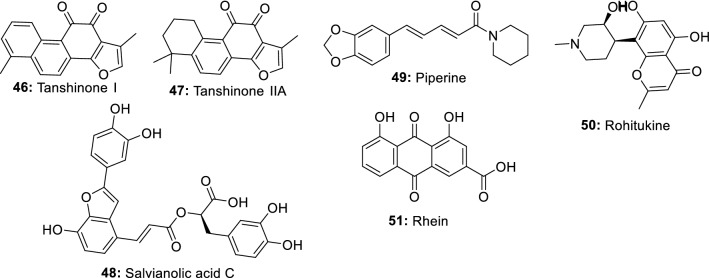
Plant anti-inflammatory metabolites also produced by endophytic fungi

In recent years, increasing researches have demonstrated the ability of endophytic fungi derived from important medicinal plants to produce the same bioactive metabolites as their host plants [56]. In this perspective, many anti-inflammatory compounds currently isolated from medicinal plants have been identified in the metabolome of endophytic fungi. We are discussing in this section a few numbers of plant anti-inflammatory compounds produced by endophytes (Fig. 2).

*Salvia miltiorrhiza* Bunge is a valuable medicinal plant used for hundreds of years for the management of several diseases including cardiovascular diseases, dysmenorrhea, amenorrhea, and hypertension, hepatocirrhosis, chronic renal failure and other diseases [57, 58]. Among its chemical constituents, tanshinones have received extensive attention over the years because of their remarkable activities in the clinical treatment of cardiovascular diseases. Additional properties such as antibacterial, antioxidant, anti-tumor and anti-inflammatory have also been intensively demonstrated [59, 60]. However, one of the difficulties of producing tanshinones from *S. miltiorrhiza* is the very low extraction yield. This situation is not helping since the clinical demand for these compounds is constantly increasing. Therefore, researches are currently exploring a new approach to produce such important molecules [60]. In this respect, eighteen endophytic fungal strains isolated from the roots of *Salvia miltiorrhizae* were screened for their ability to produce tanshinones using HPLC and LC-HRMS/MS analyses. One fungus identified as *Trichoderma atroviride* D16 was found to produce 1.119 g/g of tanshinone I (**46**) and 3.049 g/g of tanshinone IIA (**47**) [61]. In a later study, Ming et al. [62] showed that mycelium extract and the polysaccharide fraction produced by *T. atroviride* D16 stimulated the biosynthesis of tanshinones in hairy roots of *S. miltiorrhiza*. These studies demonstrated that in addition to his ability to produce tanshinones, *T. atroviride* D16 can also act as an elicitor to stimulate the large-scale production of tanshinones by *S. miltiorrhiza*. In addition to tanshinones, salvianolic acids also found in *Salvia miltiorrhiza in abundance are well known for their multiple properties including anti-inflammation* [63]. *In a study conducted to identify potential endophytes-producing salvianolic acids*, *extracts from a* total of 58 endophytic fungal strains were screened using HPLC and LC-HRMS/MS analyses. A fungus identified as *Phoma glomerata* D14 was able to produce salvianolic acid C (**48**), in both the fermentation broth and the mycelia [64].

*Piper nigrum* (Black pepper) is a plant commonly used as a spice, preservative, and medicine in many countries. This plant is well-reputed for its anti-inflammatory activities [65] which is mainly associated with piperine, the main constituent. Other pharmacological effects of piperine include antinociceptive, and antiarthritic [66, 67]. To identify a novel source to produce piperine (**49**), several endophytic fungi isolated from the stem of *Piper nigrum* were screened using HPLC and LCMS analyses. *Colletotrichum gloeosporioides* show the potential to produce piperine and can be exploited to scale up the industrial production of this compound [68].

Rohitukine, a chromone alkaloid is a natural product only found in four plant species, *Amoora rohituka*, *Dysoxylum binectariferum*, *Schumanniophyton magnificum* and *Schumanniophyton problematicum* [69]. This compound is known for its multiple properties including anti-inflammatory, anti-cancer, and immuno-modulatory. The unique scaffold of rohitukine has offered the medicinal chemists the opportunity to synthesize novel molecules. Indeed, flavopiridol and P-276-00, two novel semi-synthetic derivatives are now in an advanced stage of clinical development and trial for cancer treatment [70]. Flavopiridol recently approved as an orphan drug for the treatment of chronic lymphocytic leukemia cancer is a potent CDK inhibitor (CDK1, CDK2, CDK4, CDK6, CDK8, and CDK9) known to arrest the cell cycle at both G1/S and G2/M phases [71]. In the search for rohitukine-producing endophytic fungi, Mohana Kumara et al. [72] identified *Fusarium proliferatum* (MTCC 9690) isolated from the inner bark tissue of *Dysoxylum binectariferum* Hook.f as able to produce 186 μg/100 g dry mycelial weight of rohitukine (**50**) in vitro. In a later study, Kumara et al. [73] identified *Fusarium oxysporum* (MTCC-11383), *Fusarium oxysporum* (MTCC-11384) and *Fusarium solani* (MTCC-11385) producing 192.78 μg to 359.55 μg/100 g of dry weight and 14.10 to 71.90 μg/100 mL of rohitukine in both mycelium and broth medium respectively.

Rhein (**51**) is an anthraquinone metabolite present in many medicinal plants including *Rheum palmatum*, *Cassia tora*, *Polygonum multiflorum*, and *Aloe barbadensis*. This compound is known to have good hepatoprotective, nephroprotective, anti-cancer, anti-inflammatory, antimicrobial and hemostatic and several other protective effects. In a study to investigate the potential of microbial species to produce rhein, 14 endophytic fungi isolated from *R. palmatum* L. were screened using TLC, HPLC, and LC–MS analyses through a direct comparison with authentic rhein standard. A potent fungus identified as *Fusarium solani* could produce 5.672 mg/L [74].

Altogether, these studies demonstrate once again the significant industrial potential of endophytic fungi and suggest that proper exploitation could help meet the pharmaceutical demands for many important drugs in a cost-effective, easily accessible, and reproducible way.

#### Anti-inflammatory Agents Produced by Endophytic Fungi from Terrestrial Plants

In the past years, exploring the potential of endophytes for new anti-inflammatory agents has increased. Several studies have reported the activity of compounds produced by endophytic fungi from diverse medicinal plants. We are discussing in this section the activity of extracts and isolated compounds. Extracts from five endophytic fungi were investigated for their ability to inhibit the production of nitric oxide, CD40 phenotype and, pro- and anti-inflammatory cytokine in lipopolysaccharide (LPS)-stimulated BV2 microglia cells. From the results, the microglia pre-treated with endophytic extracts at 0.1 mg/mL significantly reduced NO production without compromising cell viability. Moreover, the expression of the pro-inflammatory cytokines, IL-6, and TNF-α in LPS-stimulated microglia were inhibited as well [75]. In another study, the ethyl acetate extract of *Penicillium crustosum* from *Phoenix dactylifera* was reported to significantly altered LDH levels and reduced IL-6 transcript expression on the MCF7 cell line using the quantitative real-time PCR technique [76].

**Fig. 3:**
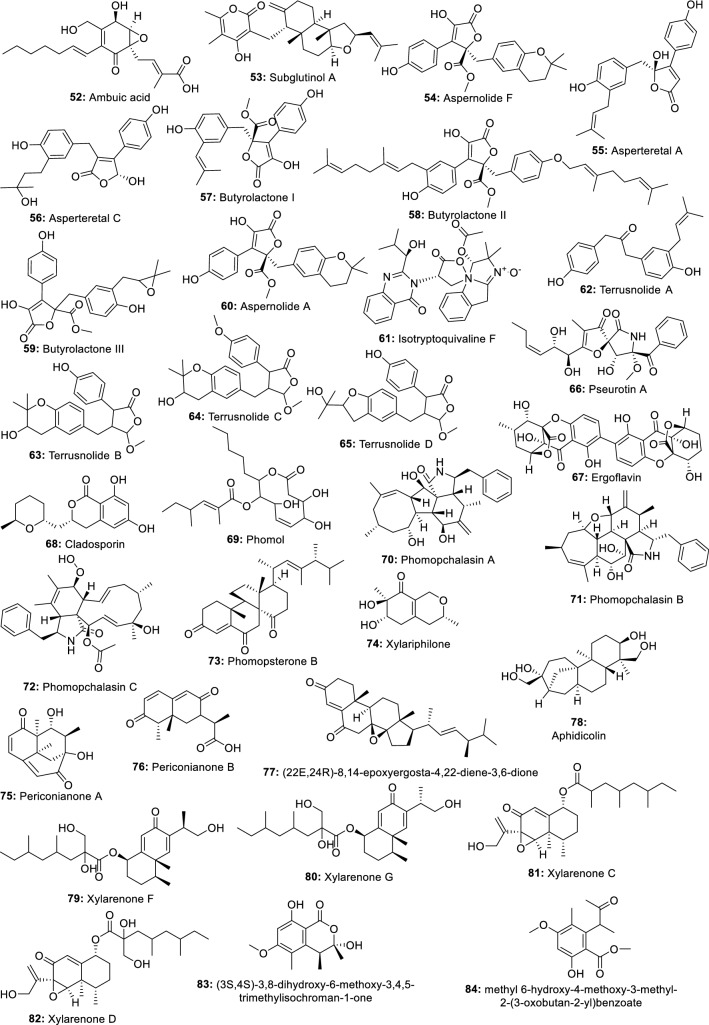

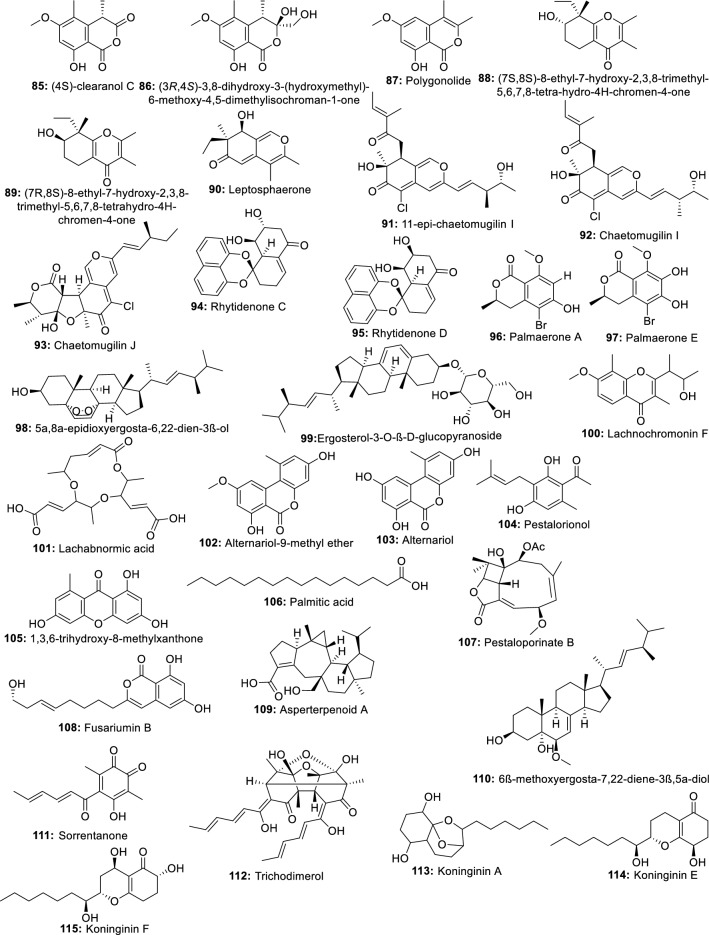
Anti-inflammatory compounds isolated from endophytic fungi of terrestrial plants

Methanol and water extracts of four endophytes isolated from different parts of *Loranthus* sp. were investigated for anti-inflammatory activity using albumin denaturation, membrane stabilization, and proteinase inhibitory assays. The results show that methanol extracts of *A. niger, Penicillium* sp. and *Alternaria alternata* inhibiting the heat-induced albumin denaturation (87.88, 86.89, and 87.03 µg/mL), the red blood cells membrane stabilization (78.42, 77.61, and 77.98 µg/mL) and the proteinase activity (85.21, 84.09, and 79.17 µg/mL) were the more potent [77]. In accordance, Shoba and Sathiavelu [78] showed that among the three solvent extracts from endophytic fungus *Cochliobolus* sp. from *Aerva lanata*, the methanol extract was the most potent exhibiting 62.51% inhibition of the egg albumin denaturation. In another investigation, the anti-inflammatory properties of the methanol extract from the endophytic fungus *Rhizoctonia* sp. were investigated in mice using a murine model of paw edema. At 10 mg/kg the extract inhibited carrageenan-induced edema as well as histamine- or PGE (2)-induced edema [79]. Similarly, methanol and ethyl acetate extracts from 27 endophytic fungi isolated from 10 Egyptian medicinal plants were screened for the production of active anti-inflammatory metabolites and against adjuvant-induced arthritis (AIA) rat model. Extracts from endophyte *Chaetomium globosum* KC811080 showed a significant reduction in the severity of arthritis 6 days after treatment, with the methanol extract scoring 10.7 compared to 13.8 for the positive control [80]. From another screening of 16 *Myrothecium* isolates from *Calophyllum apetalum* Willd, a fungus *Myrothecium* sp. M1-CA-102 exhibited promising activity with an IC_50_ value of 58 and 8 μg/mL for 15-lipoxygenase (15-LOX) and human cyclooxygenase-2 (COX-2), respectively [81]. These results from the above investigations suggest that the extracts from endophytes possess constituents with anti-inflammatory properties that may be developed as drugs against inflammatory disorders. These results serve as justification for further chemical investigations which led to the isolation of a dozen active ingredients (Fig. 3).

In a recent investigation, ambuic acid (**52**) isolated from endophytic fungus *Pestalotiopsis neglecta* was reported for its activity on lipopolysaccharide-induced inflammation in RAW264.7 macrophages. This compound exhibited a concentration-dependent suppression of the overproduction of nitric oxide and prostaglandin E2. Remarkably, ambuic acid also inhibited the release of the pro-inflammatory cytokine interleukin-6 (IL-6) without inhibiting the release of tumor necrosis factor-α. In further investigations, compound 52 also downregulated the LPS-induced high expression of inducible nitric oxide (NO) synthase (iNOS) and cyclooxygenase-2 proteins and inhibited the enzymatic activity of iNOS and COX-2. Besides, the phosphorylation of extracellular signal-regulated kinase 1/2 (ERK 1/2) and c-Jun N-terminal kinase (JNK) induced by LPS was also suppressed. From the results, the authors hypothesized that 52 may exert its anti-inflammatory action by blocking the activation of the ERK/JNK MAPK signalling pathway [82]. This compound, well-known for its antimicrobial activity is a small molecule (MW 350.4) and can be repurposed as anti-inflammatory agents or could constitute a very good starting point for the synthesis of more potent drugs. Indeed, several derivatives from ambuic acid were previously reported by Ding et al. [83] and their investigation could reveal more potent anti-inflammatory agents. Another well-known compound, subglutinol A (**53**) an immunosuppressive agent from *Fusarium subglutinans*, an endophyte of *Tripterygium wilfordii* was found to profoundly inhibit the production of pro-inflammatory IFNγ and IL-17. Further investigation shows that **53** may exert its anti-inflammatory effects by exacerbating mitochondrial damage in T cells. He also significantly reduces lymphocytic infiltration and ameliorates footpad swelling in the mouse model of Th1-driven delayed-type hypersensitivity [84]. Like ambuic acid, subglutinol A is a small molecule suitable for further medicinal chemistry for the synthesis of more active compounds. In that respect, Lee et al. [85] reported the activity of several subglutinol A derivatives against IL‐2, one of the major target cytokines in inflammatory diseases. These studies are demonstrating that metabolites from endophytes are sources of potent molecules, suitable for medicinal chemistry studies and suggest that further exploration could reveal novel anti-inflammatory leads.

An unidentified compound C produced by endophytic fungus *Talaromyces wortmannii* was reported to impede P. acnes-mediated activation of NF-κB and AP-1 by inhibiting in a dose-dependent manner the IκB degradation, the phosphorylation of ERK and JNK MAP kinases, and the release of IL-8 [86]. This result may suggest that compound C can be useful in the development of a new drug against inflammation and acne. More recently, aspernolide F (**54**) a compound isolated from the endophytic fungus *A. terreus* was evaluated in rats for its potential cardioprotective effects. From the results, **54** effectively protected against DOX-induced cardiac damage by counteracting DOX-induced ECG abnormalities. As well, **54** remarkably lower down the level of inflammatory cytokines (NO, TNF-α, and IL-6) in the cardiac tissue [87]. Collectively the anti-inflammatory activities of 54 oriented toward DOX-induced cardiac damage might suggest that further investigation of this compound could lead to new drugs to manage heart disorders caused by chronic inflammation.

In the previous study, ten metabolites including three new butenolides, asperteretal A, asperteretal B, and asperteretal C, and seven known butenolides were isolated from an extract of another *Aspergillus terreus* PR-P-2, an endophyte of *Camellia sinensis* var. assamica. Among these compounds, asperteretal A (**55**), asperteretal C (**56**), butyrolactone I (**57**), butyrolactone II (**58**), butyrolactone III (**59**) and aspernolide A (**60**) showed potent inhibitory effects on NO production in RAW 264.7 lipopolysaccharide-induced macrophages [88]. From another *Aspergillus* sp. CM9a, an endophytic fungus of *Cephalotaxus mannii*, Isotryptoquivaline F (**61**) was isolated and showed good TNF-α antagonistic [89]. Similarly, terrusnolides A-D (**62**–**65**), four new butenolides isolated from *Aspergillus* sp., endophyte of *Tripterygium wilfordii* exhibited excellent inhibitory effects on the production of interleukin-1β (IL-1β), TNF-α, and NO in LPS-induced macrophages [90]. Among the six compounds isolated from endophytic fungus *Aspergillus fumigatus*, pseurotin A (**66**) exhibited anti-inflammatory activity by suppressing the lipopolysaccharide-induced pro-inflammatory factors in BV2 microglial cells, with an IC_50_ of 5.20 µM [91]. These results indicated that more investigation into metabolites from *Aspergillus* spp. might lead to new potential anti-inflammatory lead compounds for further development.

Ergoflavin (**67**), a very good anti-inflammatory compound, was isolated from an endophytic fungus of *Mimosops elengi* using bioactivity-based fractionation [92]. Cladosporin (**68**) isolated from the endophytic fungus *Cladosporium cladosporioides* was also reported for its anti-inflammatory activities [93]. Phomol (**69**), an antimicrobial agent isolated from *Phomopsis* species endophyte of *Erythrina crista-galli*, also demonstrated an interesting anti-inflammatory activity in the mouse ear assay [94]. From another endophytic fungus *Phomopsis* sp. shj2, from the *Isodon eriocalyx var. laxiflora*, phomopchalasins A (**70**) and B (**71**), two novel cytochalasans, and phomopchalasin C (**72**) exhibiting anti-inflammatory activity were isolated [95]. Two new compounds, phomopsterones A and B were isolated from endophyte *Phomopsis* sp. TJ507A, and only phomopsterone B (**73**) showed potency [96]. Similarly, seven new compounds (annulohypoxylomans A-C, annulohypoxylomanols A and B, annulohypoxyloside, annulohypoxylomarin A and xylariphilone) were isolated from ethyl acetate extract of *Annulohypoxylon truncatum,* the endophyte of *Zizania caduciflora*. Only xylariphilone (**74**) exhibited significant inhibitory effects on LPS-induced interleukin (IL)-6, IL-12 p40, and TNF-α production with IC_50_ values of 5.3, 19.4, and 37.6 μM, respectively [97]. This compound with micromolar activity represents a good candidate for new lead discovery. Periconianone A (**75**), and periconianone B (**76**) two new metabolites exhibiting potent anti-inflammatory activity were isolated from the endophytic fungus *Periconia* sp. by Zhang et al. [98]. Based on their structures, 21 analogs were synthesized using medicinal chemistry which led to the identification of two highly potent compounds that could serve as leads for further investigation [99]. These findings are very encouraging and support the view that more potent molecules could be synthesized from active metabolites produced by endophytes.

Two other compounds (22*E*,24*R*)-8,14-epoxyergosta-4,22-diene-3,6-dione (**77**) and aphidicolin (**78**) isolated from *Papulaspora immersa* and *Nigrospora sphaerica* were found to control the inflammatory cascade through overexpression of thymosin beta 4, RhoGDI2, and 14-3-3 proteins in treated cells [100]. The investigation of extract from *Camarops* sp. endophytic fungus isolated from *Alibertia macrophylla* led to the isolation of two new eremophilane-type sesquiterpenes, xylarenones F (**79**) and G (**80**), together with the two known compounds xylarenones C (**81**) and D (**82**), all exhibiting potent anti-inflammatory activity [101]. Recently, eight compounds including two new isochromanone derivatives (3*S*,4*S*)-3,8-dihydroxy-6-methoxy-3,4,5-trimethylisochroman-1-one (83) and methyl (*S*)-8-hydroxy-6-methoxy-5-methyl-4a-(3-oxobutan-2-yl)benzoate (**84**), and six known compounds including (4*S*)-clearanol C (**85**) (3*R*,4*S*)-3,8-dihydroxy-3-hydroxymethyl-6-meth-oxy-4,5-dimethylisochroman-1-one (**86**), polygonolide (**87**), (7*S*,8*S*)-8-ethyl-7-hydroxy-2,3,8-trimethyl-5,6,7,8-tetra-hydro-4H-chromen-4-one (**88**), (7*R*,8*S*)-8-ethyl-7-hydroxy-2,3,8-trimethyl-5,6,7,8-tetrahydro-4H-chromen-4-one (**89**), and leptosphaerone (**90**) were isolated from the culture of *Phoma* sp. PF2, an endophyte of *Artemisia princeps* and all showed moderate inhibitory activities on nitric oxide levels in lipopolysaccharide-induced RAW264.7 macrophage cells [102].

The chemical investigation of an endophytic fungus *Chaetomium globosum* isolated from leaves of *Wikstroemia uva-ursi* led to the isolation of two new azaphilones, chaetoviridins J and K, along with five known derivatives. Among them, 11-*epi*-chaetomugilin I (**91**), chaetomugilin I (**92**), and chaetomugilin J (**93**), along with the derivatives, namely, chaetomugilins E and F inhibited the nitric oxide production with IC_50_ values ranging from 0.3 to 5.8 μM. Compounds **91**, **92**, and chaetomugilin F also displayed (TNF-α)-induced NF-κB activity with IC_50_ values in the range of 0.9–5.1 μM [103]. Six new compounds rhytidenones A-F were isolated from the extract of a cultured fungal endophyte, *Rhytidhysteron* sp. AS21B. Only, rhytidenone C (**94**) and rhytidenone D (**95**) exhibited significant inhibitory activity against nitric oxide production from activated macrophages with the IC_50_ values of 0.31 and 3.60 μM, respectively [104]. Similarly, seven new compounds, palmaerones A-G, along with eleven known dihydroisocoumarins, were isolated from *Lachnum palmae*, an endophytic fungus from *Przewalskia tangutica* by exposure to a histone deacetylase inhibitor SAHA. Palmaerones A (**96**) and E (**97**) were the only active, exhibiting moderate inhibitory effects on NO production in LPS-induced RAW 264.7 cells, with the IC_50_ values of 26.3 and 38.7 μM, respectively [105]. Recently, a new β-tetralonyl glucoside, methylberchemiaside, along with five known compounds were isolated from a fungus *Colletotrichum* sp. GDMU-1 endophyte of *Santalum album*. When tested for the inhibitory effects on the NO production, only 5α,8α-epidioxyergosta-6,22-dien-3β-ol (**98**) and ergosterol-3-*O*-β-d-glucopyranoside (**99**) showed moderate activity with an IC_50_ value of 30.4 and 8.9 μM, respectively [106].

The fractionation of the ethyl acetate extract of *Lachnum abnorme* Mont. BCRC 09F0006, endophyte of *Ardisia cornudentata* Mez led to the isolation of four new compounds lachnochromonins D–F and lachabnormic acid, along with nine known compounds. Among them, lachnochromonin F (**100**), lachabnormic acid (**101**), alternariol-9-methyl ether (**102**), alternariol (**103**), pestalorionol (**104**), 1,3,6-trihydroxy-8-methylxanthone (**105**), and palmitic acid (**106**) inhibited the NO production in lipopolysaccharide (LPS)-activated RAW 264.7 murine macrophages [107]. In a similar investigation, Liu et al. [108] reported that among the eight new compounds (pestaloporinates A-G and 14-acetylhumulane) isolated from endophytic fungus *Pestalotiopsis* sp., from *Melia azedarach* Linn., pestaloporinate B (**107**) was potent with IC_50_ value of 19.0 μM. The same model assay was used to reveal the activity of fusariumin B (**108**) and asperterpenoid A (**109**) (IC_50_ value of 50.3 µM and 1.6 µM respectively), the two active compounds among the six isolated from an extract of *Fusarium* sp. YD-2, an endophyte from the twigs of *Santalum album* [109]. Only 6β-methoxyergosta-7,22-diene-3β,5α-diol (**110**), sorrentanone (**111**), and trichodimerol (**112**) among the fourteen compounds isolated from endophytic fungus *Trichoderma* sp. Xy24 was active against LPS-induced NO production in BV2 microglial cells with inhibitory rates of 108.2, 100, and 75.1% respectively [110]. Koninginins A, E, and F (**113**–**115**) isolated from *Trichoderma koningii* were also reported for their anti-inflammatory activities against edema-inducing, myotoxic and enzymatic activities of the total venom of *Bothrops jararacussu* snake [111]. The results indicate that these active compounds possess structural active regions that might be used as starting points in seeking for new and specific anti-inflammatory drugs against several enzymes implicated in inflammation.

An exopolysaccharide [EPS (PS-I)], having Mw∼1.87 × 10(5) Da was produced by submerged culture of an endophytic fungus *Fusarium solani* SD5. Structural elucidation revealed the presence of terminal α-l-rhamnopyranosyl (1 → 2)-α-l-rhamnopyranosyl (1 → 4)-β-d-galactopyranosyl (1 → 4,6)-β-d-galactopyranosyl moieties in a molar ratio of nearly 1:1:3:1. This exopolysaccharide exhibited in vitro anti-inflammatory and antiallergic activity and protected protects 55% erythrocytes from induced membrane lysis [112]. Silver nanoparticles (PsAgNPs) synthesized using an extract from endophytic fungal, *Penicillium* species from *Glycosmis mautitiana* also demonstrated anti-inflammatory properties by inhibiting the activity of lipoxygenase enzyme [113].

#### Anti-inflammatory Metabolites Produced by Endophytic Fungi from Marine Plants

A dozen studies reporting the activity of metabolites from endophytes of marine plants have been reported. In fact, in a recent study, 168 fungal were isolated from leaves and stems of *Acanthus ilicifolius var. xiamenensis*. Extracts from 28 isolates were investigated for anti-inflammatory activity which led to the identification of *Phoma* sp. 2 NTOU4338, *Nodulisporium* sp. NTOU4868 and *Guignardia* sp. NTOU4871 as the more potent [114]. Previously, the chemical investigation of endophytic fungal *Phoma* sp. NTOU4195 isolated from the marine red alga *Pterocladiella capillacea* led to seven new polyketides, phomaketides A-E (**116**–**120**) and pseurotins A3 (**121**) and G (**122**), along with the known compounds FR-111142 (**123**), pseurotins A (**124**), A1 (**125**), D (**126**), and F2 (**127**), 14-norpseurotin A (**128**), α-carbonylcarbene, tyrosol, cyclo(-l-Pro-l-Leu), and cyclo(-l-Pro-l-Phe). All the compounds were active against LPS-induced NO production in RAW264.7 macrophages (IC_50_ 8.8–144.3 μM) with compound 118 being the most potent [115]. Similarly, varying degree of in vitro anti-inflammatory activity was obtained with amestolkolides A-D (four new meroterpenoids), purpurogenolide E, chrodrimanin B, and chro-drimanin A isolated from extract of the mangrove endophytic fungus *Talaromyces amestolkiae* YX1. Amestolkolide B (**129**) was the most active with an IC_50_ value of 1.6 µM [116]. Chen et al. [117] showed that among the nine compounds isolated from an extract of mangrove endophytic fungus *Ascomycota* sp. CYSK-4 from *Pluchea indica*, dichlorodiaportintone (**130**), desmethyldichlorodiaportintone (**131**), desmethyldichlorodiaportin (**132**) and dichlorodiaportin (**133**) exhibited activity by inhibiting the production of NO in LPS-induced RAW 264.7 cells with an IC_50_ value ranged from 15.8–67.2 μM. In another investigation, phochrodines C and D (**134**, **135**) from mangrove entophytic fungus *Phomopsis* sp. 33# were reported to exhibit moderate inhibition of nitric oxide production with IC_50_ values of 49.0 and 51.0 μM, respectively [118]. Using the same model assay, ascomindone D (**136**) obtained from another mangrove endophytic fungus *Ascomycota* sp. SK2YWS-L, exhibited potency with an IC_50_ value of 17 µM [119]. Similarly, Liu et al. [120] reported the activity (IC_50_ 42.3 μM) of 1,2-dehydro-terredehydroaustin (**137**), isolated from the mangrove endophytic fungus *Aspergillus terreus*.

Diaporindenes A-D (**138**–**141**) (four unusual 2,3-dihydro-1 H-indene isomers), a novel isoprenylisobenzofuran A (**142**), diaporisoindoles D and E (new isoprenylisoindole alkaloids), and tenellone D (new benzophenone derivative), together with four known compounds, were isolated from an extract of the endophytic fungus *Diaporthe* sp. SYSU-HQ3 and tested for their inhibitory effects on the production of NO in lipopolysaccharide-induced RAW 264.7 cells. From the results, compounds **139**–**142**, diaporisoindoles A (**143**) and B (**144**) were found active with IC_50_ values ranging from 4.2 to 9.0 μM [121]. In the same assay model, Cui et al. [122], reported the potency of 7-deoxy-7,14-didehydrosydonol (**145**) (IC_50_ 12.5 μM), the only active metabolites among the eight compounds isolated from mangrove endophytic fungus *Aspergillus versicolor* SYSU-SKS025. Likewise, lasiodiplactone A (**146**), another moderate inhibitor (IC_50_ 23.5 μM) with unprecedented lactone, was obtained from the mangrove endophytic fungus *Lasiodiplodia theobromae* ZJ-HQ1 [123]. The chemical investigations of extracts of *Gaeumannomyces* sp. JS0464 endophyte of *Phragmites communis* led to the isolation of two glycosylated dialkylresorcinol derivatives, an anthraquinone derivative and eight known compounds among which stemphol C (**147**), stemphol D (**148**), 1-*O*-methyl-6-*O*-(α-d-ribofuranosyl)-emodin (**149**), 1-*O*-methylemodin (**150**),2-(4-hydroxyphenyl) acetic acid (**151**), cycloartenyl ferulate (**152**), ergosterol peroxide, 5α,8α-epidioxy-(22*E*,24* R*)-23-methylergosta-6,22-dien-3β-ol, and β-sitosterol were active [124]. In another investigation, three new sesquiterpenoids, trichoacorenols B-C and cyclonerodiol B, along with three known ones, isolated from the mangrove plant endophytic fungus *Trichoderma* sp. Xy24 were reported to exhibit varying degree of anti-inflammatory activity with cyclonerodiol B (**153**) and (1*R*,2*R*,4*S*,5*R*,7*S*)-4-isopropyl-1,8-dimethylspiro[4.5]dec-8-ene-2,7-diol (**154**) being more potent than curcumin, used as positive control [125].

**Fig. 4:**
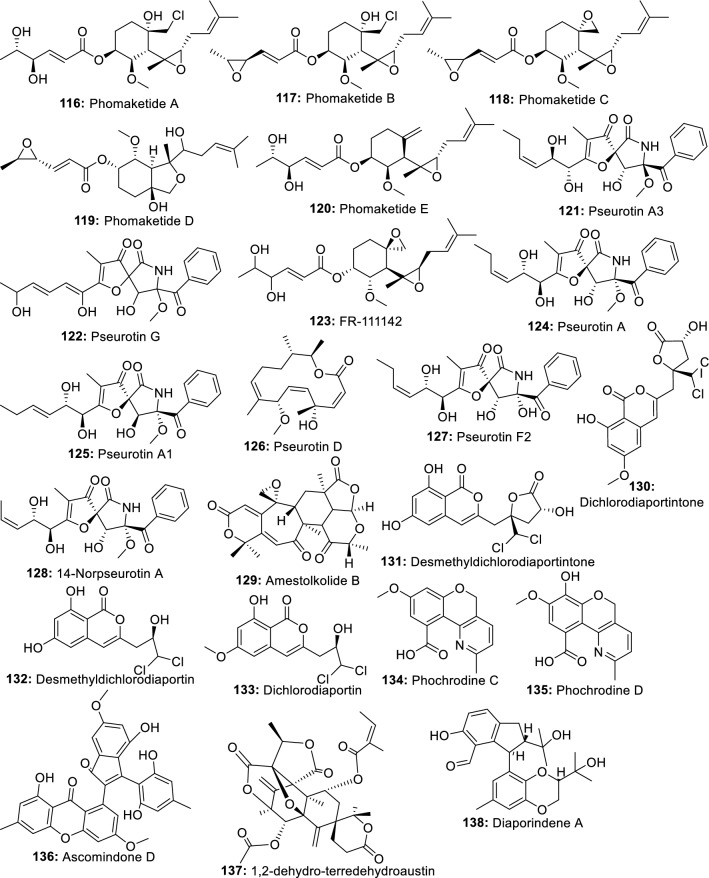

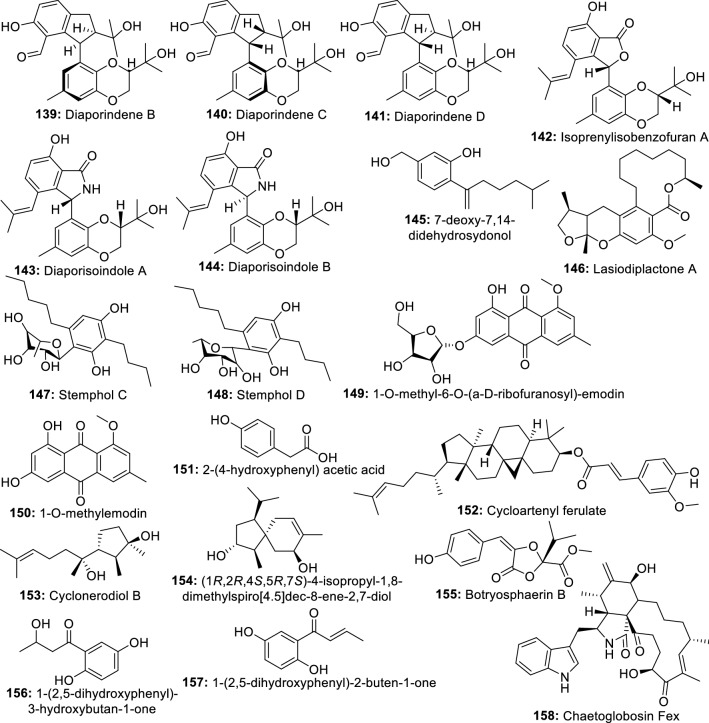
Selected active metabolites produced by endophytic fungi from marine plants

Seven compounds were isolated from an extract of endophytic fungus *Botryosphaeria* sp. SCSIO KcF6, among which, only botryosphaerin B (**155**) exhibited a specific COX-2 inhibition with an IC_50_ value of 1.12 μM [126]. Nine compounds were isolated from an extract of the marine endophytic fungus *Paraconiothyrium* sp. VK-13. Among these compounds, 1-(2,5-dihydroxyphenyl)-3-hydroxybutan-1-one (**156**), and 1-(2,5-dihydroxyphenyl)-2-buten-1-one (**157**) inhibited the overproduction of pro-inflammatory mediators NO and PGE2 in LPS-stimulated RAW264.7 cells, with IC_50_ values ranging from 3.9 to 12.5 µM. Further analysis revealed that these compounds significantly induce the suppression of iNOS and COX-2 protein expression. Furthermore, both compounds inhibited the mRNA expression of TNF-α, IL-1β, IL-6, and IL-12, with IC_50_ values ranging from 2.4–12.5 µM [127]. Similarly, chaetoglobosin Fex (**158**), a compound isolated from marine-derived endophytic fungus *Chaetomium globosum* QEN-14 was also reported to significantly inhibit the production of TNF-α, IL-6 and monocyte chemotactic protein-1 (MCP-1) in peritoneal macrophages and murine macrophage cell line RAW264.7. Compound **158** also down-regulated the mRNA expressions of several pro-inflammatory cytokines. From the analysis of data, the authors suggested that the anti-inflammatory property of 158 may be attributed to NF-κB inhibition as well as the negative regulation of ERK1/2, p38, and JNK1/2 phosphorylations [128]. The results suggest that endophytes from mangrove plants could be potential resources for bioactive natural products that can be developed as new potent and less toxic anti-inflammatory drugs (Fig. 4).

#### Metabolites from Endophytic Bacteria

**Fig. 5:**
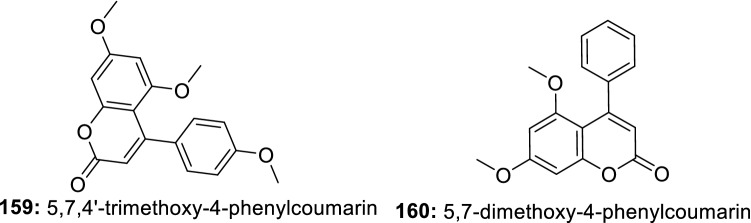
Anti-inflammatory compounds isolated from endophytic bacteria

In a course of searching for novel anti-inflammatory agents, the in vitro anti-inflammatory action of 5,7,4′-trimethoxy-4-phenylcoumarin (**159**) and 5,7-dimethoxy-4-phenylcoumarin (**160**) (Fig. 5) isolated from *Streptomyces aureofaciens* CMUAc130, an endophyte of the root of *Z. officinale* was investigated. The results demonstrated that both compounds inhibited the expression of iNOS and COX-2 protein and significantly reduced the formation of TNF-α [129]. These two small molecule-like compounds previously reported for their antimicrobial activity [130] could constitute a good starting point for the synthesis of more potent compounds with a broad activity spectrum.

## Conclusion and Perspectives

Today, leishmaniases are one of the major causes of death in developing countries while chronic inflammatory diseases are life-threatening in both developing and developed countries. The exploration of plant endophytes constitutes a very good strategy for finding new drugs against these diseases. Looking at data from our investigation, dozens of new metabolites active against different Leishmania parasites have already been reported from endophytes. Because of their novelty and structural diversity, these metabolites can be further investigated as starting points for new drug discovery against leishmaniases. Using medicinal chemistry, these metabolites can also be used to create libraries of natural and semi-synthetic compounds to promote the discovery of new drugs against several other neglected diseases. This is of great importance since the drug discovery against neglected tropical diseases is facing several drawbacks today among which the lack of diversified compounds libraries.

However, our analysis of available data also shows that a very limited number of studies on the potential of endophytes as a source of anti-leishmanial metabolites have been reported so far. The investigations reported were conducted mostly in countries such as Brazil, Panama, Egypt, and Pakistan. This means that endophytes from medicinal plants growing in diverse habitats (terrestrial and marine) of other endemic regions of Asia and Africa in general, still need to be investigated. It also appears that among endophytic organisms, limited genera of fungi have been investigated so far. Moreover, except for the report by Rahman et al. [131], demonstrating the activity of extract from *Enterobacter cloaca* endophyte of *Fagonia indica* against *Leishmania tropica* (IC_50_ value of 1.4 µg/mL), no information is available regarding the antileishmanial activity of extracts and metabolites from bacteria and actinomycetes endophytes. Overall, endophytes are still untapped sources of anti-leishmanial compounds waiting to be harness for the benefit of mankind.

Our analysis of literature data concerning anti-inflammatory activity shows that many active metabolites have already been reported from endophytic fungi. Most of these active compounds are suitable for further investigation because of the low micromolar activity, structural novelty, and their small molecular weight. This can be supported by studies of Lee et al. [85] who reported the synthesis of more potent anti-inflammatory compounds from subglutinol A or Jain et al. [70] reporting the synthesis of new anticancer drugs from rohitukine.

This investigation also shows that mainly fungi have been investigated so far and almost inexistent are reports regarding the anti-inflammatory potential of bacteria and actinomycetes endophytes. Moreover, apart from the study by Mahapatra and Banerjee [112] regarding the anti-inflammatory activities of exopolysaccharide from endophyte, no other information is available regarding the potential of this group of metabolites. This is also true for the potential green nanoparticles derived from endophytes extracts.

Overall, to fill the drug discovery pipeline with new chemical scaffolds, more investigations of these microbial factories of active metabolites are urgently needed. It is clear from our observation that there is an important imbalance of data regarding the exploration of fungi, bacteria and actinomycetes endophytes as source of antileishmanial and anti-inflammatory metabolites. Therefore, bridging this huge gap in the literature could help boost the discovery of new drugs against leishmaniases and inflammatory diseases.
